# AN INTERACTION EFFECT OF INTERNET USE AND SOCIAL PARTICIPATION ON LITERACY ABOUT FRAILTY IN OLDER JAPANESE

**DOI:** 10.1093/geroni/igad104.3178

**Published:** 2023-12-21

**Authors:** Natsumi Fujiwara, Yuya Akagi, Hiroko Yoshida, Kei Kamide, Hanayo Koetaka, Michiko Kido, Yaya Li, Mai Kabayama

**Affiliations:** Osaka University, Suita, Osaka, Japan; Osaka University, Suita, Osaka, Japan; Osaka University, Suita, Osaka, Japan; Osaka University, Suita, Osaka, Japan; Osaka University, Suita, Osaka, Japan; Osaka University, Suita, Osaka, Japan; Osaka University, Suita, Osaka, Japan; Osaka University, Suita, Osaka, Japan

## Abstract

**Objectives:**

This study aimed to analyze the age difference of the interaction effect of internet use and social participation on literacy about frailty in older Japanese.

**Methods:**

Data were collected from community-dwelling older adults aged 65-89 years randomly selected from residential registration by self-reported postal questionnaires (n=5005, response rate 49.0%). We asked them about their background, whether they use the internet to obtain health information, and whether they know the term of frailty（know what it meant / don’t know what it means but have heard of it or never heard of it and don’t know）.Logistic regression stratified by age (65-74 and 75-89 years) was used to evaluate the association of literacy about frailty with internet use and social participation adjusted for gender,education, frailty status, physical activity. The interaction between internet use and social participation on literacy about frailty was also assessed.

**Results:**

Results showed that in both age groups, the interaction revealed that a combination of social participation and internet use was associated with a high literacy about frailty (65-74 years: OR=1.64,95%CI=1.28-2.11; 75-89 years: OR=1.92,95%CI=1.45-2.56). Only in the 75-89 age group, Internet use alone without social participation was significantly associated with a high literacy about frailty (OR=1.60,95%CI=1.12-2.30).

**Conclusion:**

Our findings suggest that internet use may be beneficial for improving literacy about frailty especially for the elderly over 75 years old, however, social participation should also be taken into consideration for those. Longitudinal research is needed.

